# Dysregulated Tim-3 expression on natural killer cells is associated with increased Galectin-9 levels in HIV-1 infection

**DOI:** 10.1186/1742-4690-10-74

**Published:** 2013-07-18

**Authors:** Stephanie Jost, Uriel Y Moreno-Nieves, Wilfredo F Garcia-Beltran, Keith Rands, Jeff Reardon, Ildiko Toth, Alicja Piechocka-Trocha, Marcus Altfeld, Marylyn M Addo

**Affiliations:** 1 Ragon Institute of MGH, MIT and Harvard, 400 Technology Square, Cambridge, MA 02139, USA; 2 Heinrich-Pette-Institut, Leibniz Institute for Experimental Virology, Hamburg, Germany

**Keywords:** Tim-3, Gal-9, HIV-1, Innate immunity, NK cells

## Abstract

**Background:**

Natural killer (NK) cells constitutively express high levels of Tim-3, an immunoregulatory molecule recently proposed to be a marker for mature and functional NK cells. Whether HIV-1 infection modulates the expression of Tim-3 on NK cells, or the levels of its ligand Galectin-9 (Gal-9), and how signaling through these molecules affects the NK cell response to HIV-1 remains inadequately understood.

**Results:**

We analyzed Tim-3 and Gal-9 expression in a cohort of 85 individuals with early and chronic HIV-1 infection, and in 13 HIV-1 seronegative control subjects. HIV-1 infection was associated with reduced expression of Tim-3 on NK cells, which was normalized by HAART. Plasma concentrations of Gal-9 were higher in HIV-1-infected individuals than in healthy individuals. Interestingly, Gal-9 expression in immune cells was significantly elevated in early infection, with monocytes and dendritic cells displaying the highest expression levels, which correlated with HIV-1 viral loads. *In vitro*, Gal-9 triggered Tim-3 downregulation on NK cells as well as NK cell activation.

**Conclusions:**

Our data suggest that high expression levels of Gal-9 during early HIV-1 infection can lead to enhanced NK cell activity, possibly allowing for improved early control of HIV-1. In contrast, persistent Gal-9 production might impair Tim-3 activity and contribute to NK cell dysfunction in chronic HIV-1 infection.

## Background

The human immunodeficiency virus type 1 (HIV-1) affects 34 million adults and children worldwide, and the ongoing spread of the epidemic resulted in about 2.5 million new infections and 1.7 million deaths in 2011 alone [1]. Novel approaches to prevent the transmission of HIV-1 are urgently needed, and whether manipulating innate immune effector cell function could be used as a strategy to enhance HIV-1 vaccine efficiency remains to be determined. Recent data suggest that incorporating components with the potential to harness the antiviral function of natural killer (NK) cells may represent an attractive option to improve future vaccine designs [2-13].

NK cells play an important role in containing viral replication in the very early stages of viral infections, and in shaping the subsequent adaptive immune response by interacting with other immune cells, including dendritic cells (DCs) and CD4+ T cells [14-17]. NK cell function is regulated by the integration of inhibitory and activating signals generated by an arsenal of cell surface receptors, with effector functions taking place when activating signals overcome inhibitory ones [18]. Importantly, mounting evidence suggests that NK cells can mediate antiviral activity in HIV-1-infected humans [2-12]. However, how NK cells recognize and eliminate HIV‒1‒infected cells and control HIV‒1 replication and disease progression remains poorly understood to date.

It has recently been described that NK cells constitutively express high levels of T-cell immunoglobulin and mucin domain-containing molecule 3 (Tim-3) [19-21]. Tim-3 is a type I transmembrane receptor that is also expressed on specific subsets of CD4+ and CD8+ T cells, on subpopulations of macrophages and DCs, and on monocytes (reviewed in [22]), albeit to a lesser extent than on NK cells [19]. Tim-3 was originally identified as a marker of terminally differentiated CD4+ Th1 cells [23], and subsequently associated with T-cell exhaustion and impaired virus-specific T-cell responses in HIV-1, hepatitis C virus (HCV) and hepatitis B virus (HBV) infection [24-28]. To date, three ligands have been described for Tim-3, including Galectin-9 (Gal-9), a 40-kD S-type β-galactoside-binding lectin [29], cell-surface phosphatidylserine [30,31], and—at least in murine models—the high-mobility group box 1 (HMGB1) protein [32]. Gal-9 is highly expressed in immune tissues [33], and while engagement of Tim-3 triggers apoptosis in CD4+ Th1 cells [29], T cells and thymocytes [34], this interaction has been suggested to (i) protect activated CD4+ T cells from HIV-1 infection and replication [35], (ii) enhance the production of pro-inflammatory cytokines by immature dendritic cells [36,37] and (iii) regulate the migration of Th2 cells [38]. Therefore, Tim-3 signaling on immune cells can trigger either inhibitory or activating signals, with the outcome depending on the presence of described and potentially as yet undefined ligands for Tim-3 [39]. While Tim-3 has been extensively studied on T cells, much less is known about the impact of Tim-3-mediated signaling on NK cell responses, notably in the context of viral infections.

Expression of Tim-3 can be induced on NK cells by various cytokines [20,21], and Tim-3 has recently been proposed to be a marker for mature and fully functional NK cells, with those expressing the highest levels of the receptor displaying the most potent cytotoxic activity or cytokine production [21]. On NK cells, Tim-3 can act as an activating co-receptor, as exposure to Gal-9 enhances IFN-γ production by Tim-3+ NK cells [20], yet it can also deliver inhibitory signals, given that the ability of NK cells to kill target cells is lost upon Tim-3 cross-linking [21]. In accordance with the latter observation, up-regulation of Tim-3 on NK cells has been associated with reduced anti-viral properties in chronic hepatitis B infection, with NK cell cytolytic function being enhanced upon Tim-3 blockade [40].

Progressive HIV-1 infection leads to significant changes in NK cell phenotype and function, and is associated with an expansion of anergic NK cells [41,42]. However, it is not known whether chronic HIV-1 infection modulates the expression of Tim-3 on NK cells or the levels of its ligand Gal-9, nor is it understood how signaling through these molecules affects the NK cell response to HIV-1. Here, we describe dysregulated expression of Tim-3 on NK cells in HIV-1 infection, with differential responses in early versus chronic HIV-1 infection. Plasma levels of Gal-9 were elevated in HIV-1-infected individuals and correlated with markers of HIV-1 disease progression. Moreover, we show that stimulation of Tim-3+ NK cells by Gal-9 leads to NK cell activation and Tim-3 downregulation *in vitro.* Our data suggest that persistent signaling through Tim-3 on NK cells might result in loss of functionality and accumulation of a Tim-3^low^ NK cell population *in vivo*, thereby contributing to the previously described impaired activity of NK cells in chronic HIV-1 infection.

## Results

### HIV-1 infection is associated with decreased percentages of Tim-3+ NK cells

**Figure 1:**
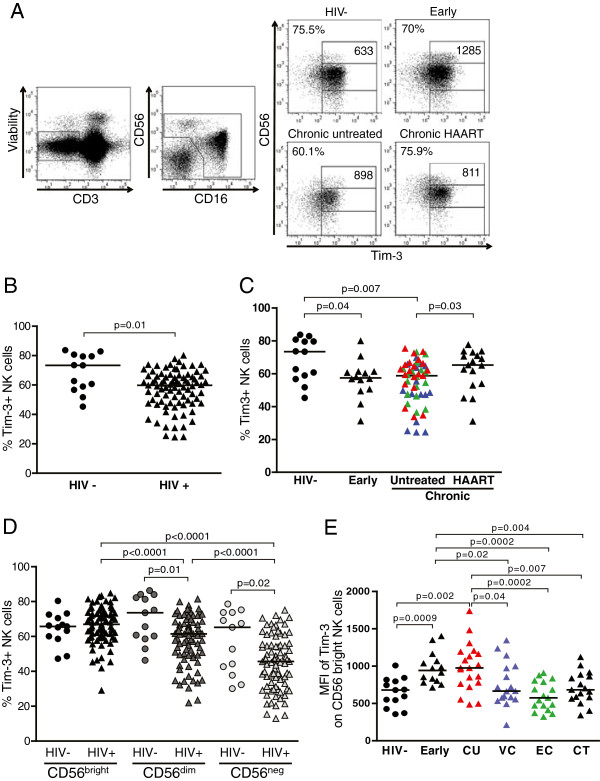
**Tim-3 expression is dysregulated in HIV-1 infection. (A)** Flow cytometry gating strategy: Gates are set to include CD3^-^ lymphocytes and exclude dead cells (viability marker^+^). NK cell subsets are defined by the expression of CD56 and CD16, and Tim-3+ NK cells by using appropriate fluorescence-minus-one controls. The four right flow cytometry panels show representative examples of Tim-3+ bulk and CD56^bright^, CD56^dim^ and CD56^neg^ NK cells in an HIV-1 negative subject (HIV-), an individual with late primary untreated infection, an untreated chronic HIV-1 progressor, and a HAART-treated HIV-1+ patient. Percentages of Tim-3+ bulk NK cells, and MFI of Tim-3 on CD56^bright^ NK cells are indicated on the top left of the main panel and on the top right of the CD56^bright^ NK cell gate, respectively. **(B)** Dot plots represent the percentage of Tim-3+ NK cells from 13 healthy individuals and 85 HIV-1-infected subjects **(C)**, including 14 with early untreated HIV-1 infection, 54 with chronic untreated HIV-1 infection (blue, 17 viremic controllers; green, 17 elite controllers; red, 20 untreated progressors), and 17 with HAART-treated HIV-1 infection. **(D)** Percentages of Tim-3 + NK cells in CD56^bright^ (CD3^-^CD56^+^CD16^-^), CD56^dim^ (CD3^-^CD56^+^CD16^+^) and CD56^neg^ (CD3^-^CD56^-^CD16^+^) NK cells in 13 HIV-1 negative and 85 HIV-1-infected subjects. **(E)** MFI of Tim-3 on CD56^bright^ NK cells in 13 healthy individuals and 14 subjects with early infection, 54 with chronic untreated HIV-1 infection, including 17 viremic controllers (blue, VC), 17 elite controllers (green, EC), 20 untreated progressors (red, CU), and 17 with HAART-treated HIV-1 infection (CT). Horizontal lines indicate the median percentages. Statistical differences with *p* < 0.05 are indicated and were determined using the Mann–Whitney t test to compare phenotype frequencies between two groups.

The immunoregulatory molecule Tim-3, previously associated with T cell exhaustion and anergy, was recently shown to mark NK cell maturation and to suppress cell-mediated toxicity [21]. As progressive HIV-1 infection leads to the accumulation of dysfunctional NK cells [41,42], we first examined whether surface expression of Tim-3 protein on NK cells was altered in individuals at different stages of HIV-1 infection when compared to healthy subjects (Table 1). As demonstrated in previous studies, our data highlight a high degree of variation between the frequencies of Tim-3-expressing NK cells in HIV-1 negative individuals (median, 73.36; interquartile range [IQR], 57.80-80.14) [21] (Figure 1). Similar heterogeneity of Tim-3 expression on NK cells was observed in HIV-1-infected individuals (median, 59.77; IQR, 47.80-66.04), yet percentages of Tim-3+ NK cells were significantly decreased in HIV-1 infection compared to healthy subjects (Figure 1A and B). Cell surface density of Tim-3 on NK cells, as measured by the mean fluorescence intensity (MFI), trended to be lower in HIV-1 infection compared to HIV-1-negative controls, however the difference did not reach statistical significance (HIV-1-infected subjects: median, 497; IQR, 387–622; healthy controls: median, 600; IQR, 457.3-805.5; p = 0.1). A substantial decrease in the number of NK cells expressing Tim-3 was observed as early as in primary HIV-1 infection, and appeared to be partially restored by highly active antiretroviral therapy (HAART) (Figure 1C). The lowest proportions of Tim-3+ NK cells were found in viremic controllers (p = 0.002 and p = 0.02 compared to healthy controls and untreated progressors, respectively), yet there was an overall high degree of variability in Tim-3 expression among these subjects (median, 48; IQR 38.7-64.3), which could not be attributed to differences in any of the demographic or clinical data analyzed (data not shown). Interestingly, elite controllers had similar percentages of Tim-3+ NK cells (median, 58.2; IQR, 47–62.7) as untreated subjects with chronic viremic HIV-1 infection (median, 62, IQR, 55.1-67).

**Table 1:** Characteristics of HIV-1-infected study participants and healthy controls

**Cohort**	**N**	**Median viral load (RNA cop/mL)**	**IQR viral load (RNA cop/mL)**	**Median CD4+ cell count (cells/mm3)**	**IQR CD4+ cell count (cells/mm3)**
**HIV negative**	13				
**Early infection**	14	51200	1383-142500	502	425-688
**Chronic untreated**	20	8137	5664-21175	547	454-712
**Chronic treated**	17	<50		558	390-916
**Elite controllers**	17	<50		803	654-1224
**Viremic controllers**	17	382	193-884	581	452-843

*IQR* interquartile range.

Functionally distinct NK cell subsets can be defined based on the level of CD56 and CD16 co-expression on NK cells. Consequently, we assessed whether Tim-3 expression differed between CD56^bright^CD16^-^, CD56^dim^CD16^+^, and CD56^neg^CD16^+^ NK cell subsets. The observed decrease in Tim-3+ NK cells in HIV-1-infected individuals was mainly driven by a decline in CD56^dim^ and CD56^neg^ NK cells expressing Tim-3, whereas no change was observed in the CD56^bright^ compartment (Figure 1D). As observed for bulk NK cells, decreased percentages of Tim3+ CD56^dim^ and CD56^neg^ NK cells were found in patients with early (p = 0.04 and not significant, respectively) and untreated chronic infection, (i.e. untreated progressors and controllers) (p = 0.008 and 0.01, respectively) (Additional file 1). While proportions of Tim-3+ CD56^bright^ NK cells did not vary between healthy controls and HIV-1-infected individuals, this subset of NK cells expressed significantly higher levels of Tim-3 during the early phase of the infection and in individuals with untreated progressive infection compared to CD56^bright^ NK cells in states of suppressed viremia, such as in HIV controllers, HAART-treated HIV-infected individuals and healthy subjects (Figure 1A and E). Increased expression of Tim-3 on NK cells might be partly explained by elevated percentages of Tim-3+ CD56^bright^ NK cells in early and progressive infection (Additional file 1).

CD4+ T-cell counts positively correlated with the proportions of bulk (p = 0.04, Spearman r = 0.28) and CD56^dim^ (p = 0.03, Spearman r = 0.31) NK cells expressing Tim-3 in subjects with untreated chronic progressive and controlled HIV-1 infection (Additional file 2). In untreated subjects with chronic HIV-1 infection and active HIV replication (i.e. detectable viral loads), percentages of Tim-3+ bulk (p = 0.006, Spearman r = 0.46) and CD56^dim^ (p = 0.004, Spearman r = 0.48) NK cells were even more strongly associated with CD4+ T-cell counts. No statistically significant associations were observed with HIV-1 viral loads (data not shown).

Taken together, untreated HIV-1 infection was associated with an early and persisting decrease in percentages of circulating CD56^dim^ and CD56^neg^ NK cells expressing Tim-3. Loss of Tim-3+ NK cells over time was more pronounced in untreated subjects (i.e. untreated chronic progressors and controllers) with low CD4+ T cell counts. Tim-3 was transiently upregulated on the immunoregulatory CD56^bright^ subset of NK cells during the early phase of HIV-1 infection, possibly reflecting a cytokine-induced maturation process as previously demonstrated *in vitro*[21]. High surface expression of Tim-3 on NK cells persisted on CD56^bright^ NK cells in untreated individuals with progressive HIV infection, while no difference was observed in individuals with low or no viremia present (HAART-treated subjects, controllers and healthy individuals).

### High expression of Galectin-9 in primary HIV-1 infection

**Figure 2:**
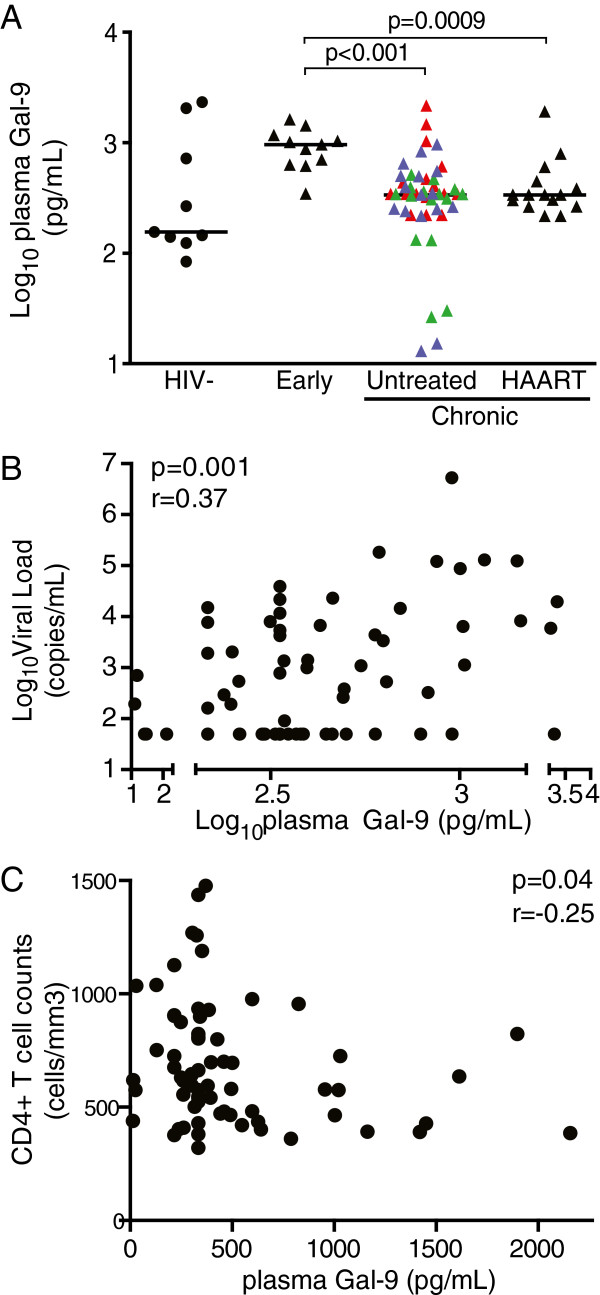
**High plasma Galectin-9 concentration in early HIV-1 infection. (A)** Dot plots represent the logarithmic concentration of soluble Gal-9 in the plasma of 9 healthy individuals, 11 subjects with early untreated HIV-1 infection, 15 with HAART-treated HIV-1 infection, and 47 untreated individuals with chronic HIV-1 infection, including 16 viremic controllers (blue), 15 elite controllers (green), and 16 subjects with chronic untreated progressive HIV-1 infection (red), as determined by ELISA. **(B)** Positive correlation between the logarithmic viral load and the logarithmic concentration of soluble Gal-9 in the plasma of 73 HIV-1 individuals, including 11 subjects with early infection, 16 viremic controllers, 15 elite controllers, 16 subjects with chronic untreated, and 15 with treated HIV-1 infection. **(C)** Inverse correlation between the CD4+ T cell counts and the concentration of soluble Gal-9 in the plasma of 69 HIV-1 individuals, including 7 subjects with early infection, 16 viremic controllers, 15 elite controllers, 16 subjects with chronic untreated, and 15 with treated HIV-1 infection. CD4-T cell counts were not available for 4 subjects with early HIV-1 infection at the exact date of the sample draw.

To further dissect the potential mechanisms leading to alterations in Tim-3 expression on NK cells, we examined whether differential plasma concentration or cellular expression of Gal-9, a Tim-3 ligand, accounts for the changes observed in early and chronic HIV-1 infection. Plasma Gal-9 levels were more than 6-times higher in late primary HIV-1 infection and twice higher in chronically infected HIV-1-positive subjects than in healthy controls (Figure 2A). There was no difference in soluble Gal-9 levels between HAART-treated and untreated subjects with progressive or controlled HIV infection. Likewise, the differences between HIV-negative and HIV-1-positive populations did not reach statistical significance. Elevated concentrations of Gal-9 in HIV-1-infected subjects were positively correlated with viremia (Figure 2B) and negatively correlated with CD4+ T-cell counts (Figure 2C).

**Figure 3:**
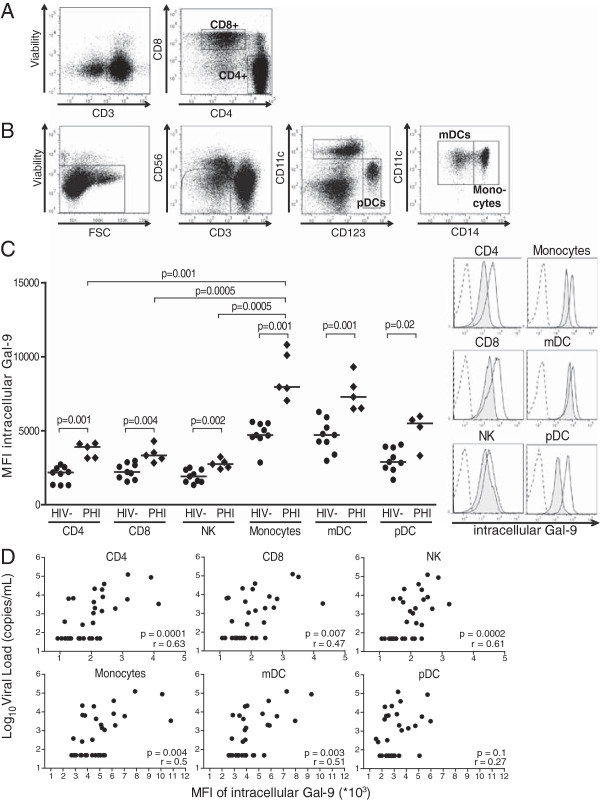
**Expression of galectin-9 in PBMCs is increased in early HIV-1 infection and correlates with HIV-1 viremia.** Flow cytometry gating strategy to analyze intracellular expression of Gal-9 on **(A)** CD4+ and CD8+ T cells and **(B)** pDCs, monocytes or mDCs. Gates are set to exclude dead cells (viability marker^+^), and expression of Gal-9 on various lymphocyte subsets is defined by using appropriate fluorescence-minus-one controls. **(C)** Dot plots display the MFI of intracellular Gal-9 in the indicated immune cell subsets from 9 healthy (HIV-) and 5 untreated subjects with early infection as determined by flow cytometry. Horizontal lines indicate the median percentages. Differences where *p* < 0.05 are indicated. Histogram overlays display representative examples of intracellular Gal-9 expression in each lymphocyte subset analyzed in healthy controls (tinted) and in individuals with early HIV-1 infection (transparent) compared to the FMO (dotted line). PHI, early HIV-1 infection **(D)** Correlations between the logarithmic viral load and the MFI of intracellular Gal-9 in CD4 + T cells, CD8+ T cells, NK cells, monocytes, mDCS and pDCs from a subset of 32 HIV-1-positive individuals, including 4 subjects with early infection, 6 viremic controllers, 6 elite controllers, 9 subjects with chronic untreated, and 7 with chronic treated HIV-1 infection.

Gal-9 is produced by a wide range of epithelial and immune cells [29]. In order to identify the predominant source of cellular Gal-9, we assessed its intracellular levels by flow cytometry in different immune cell subsets, including CD4+ and CD8+ T lymphocytes, NK cells, monocytes, myeloid and plasmacytoid dendritic cells (mDCs and pDCs, respectively) in a subset of 32 HIV-positive and 9 HIV negative controls (Figure 3A and B). In healthy individuals, Gal-9 trended to have higher intracellular expression in monocytes and DCs than in the other lymphocyte subsets examined (Figure 3C). However, in individuals with early HIV-1 infection, intracellular Gal-9 levels were significantly upregulated in all analyzed peripheral blood mononuclear cells (PBMCs), with monocytes and DCs displaying the highest expression levels. In contrast, intracellular Gal-9 levels in immune cells from subjects with chronic HIV-1 infection were similar to those in healthy individuals (data not shown). In line with the correlation observed between plasma Gal-9 levels and viral load, intracellular levels of Gal-9 in CD4+ T cells, CD8+ T cells, NK cells, monocytes, and mDCs all positively correlated with viral load in subjects with HIV-1 infection (Figure 3D). This association was maintained even after exclusion of elite controllers from the analysis (data not shown). Thus, levels of the soluble form of Gal-9 were overall increased in the plasma of HIV-1-infected individuals, specifically during the early phase of HIV-1 infection, coinciding with the early upregulation of Gal-9 in all PBMC subsets analyzed. Cellular expression levels of Gal-9 also correlated with the aforementioned parameters of disease progression.

### Exposure to soluble Gal-9 triggers NK cell degranulation and Tim-3 downmodulation

**Figure 4:**
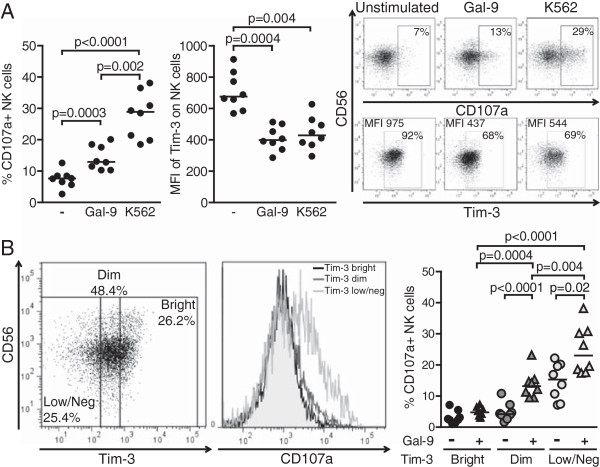
**Incubation with soluble Gal-9 increases NK cell function and decreases surface expression of Tim-3. (A)** Dot plots represent the percentages of CD107a + NK cells and the MFI of Tim-3 on NK cells from 8 healthy individuals upon pre-activation with 1 ng/mL of IL-15 and IL-18 overnight followed by stimulation with either 0.9 μg/mL of soluble Gal-9 for another 16 h or K562 target cells at an effector:target ratio of 10:1 for 6 h. Representative primary flow cytometry panels show CD107a upregulation (upper panel) and Tim-3 expression (lower panel) on NK cells that were left unstimulated, or were activated as indicated. **(B)** Unstimulated or Gal-9-activated NK cells were divided into Tim-3^bright^, Tim-3^dim^ and Tim-3^low/neg^ so that the bright and the low/neg subpopulations each consistently represents about 25% of the bulk NK cells, and subsequently analyzed for CD107a expression. Representative primary flow panel shows an example of subdivision of NK cells according to Tim-3 expression. Percentages of positive NK cells and median fluorescence intensity are indicated. Histograms display CD107a upregulation in each subset following incubation with soluble Gal-9. Horizontal lines indicate the median percentages. Statistically significant difference reached when *p* < 0.05 is indicated.

Cross-linking of Tim-3 has been shown to inhibit NK cell cytotoxic functions *in vitro*[21]. Based on our observation of increased levels of Gal-9 in natural HIV-1 infection, we sought to next investigate whether Gal-9 could modulate NK cell function *in vitro.* To address this question, we compared NK cell function following treatment with soluble Gal-9 to that upon exposure to major histocompatibility complex (MHC)-deficient target cells (i.e. K562 cells). Incubation with Gal-9 triggered NK cell activation, as measured by CD107a surface expression, which occurred concomitantly with a decreased surface expression of Tim-3 (Figure 4A). Percentages of Tim-3+ NK cells were also diminished upon Gal-9 stimulation, although to a lesser extent (data not shown). Treatment with soluble Gal-9 did not lead to a significant increase in production of IFN-γ (unstimulated: median, 0.7; IQR 0.42-0.94; Gal-9; median, 1.014; IQR, 0.3893- 2.033), as compared to incubation with K562 cells (median, 9.7; IQR, 5.08- 17.13; p = 0.008 vs. unstimulated and p = 0.004 vs. Gal-9). In order to further understand the function executed by Tim-3 in the NK cell response to Gal-9, we analyzed changes in CD107a expression on NK cells bearing high (Tim-3^bright^), medium (Tim-3^dim^) or low/no (Tim3^low/neg^) levels of Tim-3, and observed that following incubation with Gal-9, CD107a upregulation on Tim3^low/neg^ NK cells was enhanced compared to that of Tim-3^bright^ and Tim3^dim^ NK cells (Figure 4B). Tim-3^bright^ NK cells were enriched in CD56^bright^ NK cells which might intrinsically have defective degranulation properties. Therefore, we quantified CD107a upregulation on Tim-3^bright^, Tim-3^dim^ and Tim-3^low/neg^ CD56^dim^ NK cells, and found that upon Gal-9 stimulation, the activity of CD56^dim^ NK cells expressing high amounts of Tim-3 was significantly reduced compared to those expressing dim (p < 0.0001) and low (p < 0.0001) levels, showing that CD56^bright^ NK cells were not introducing a bias in our findings (data not shown).

Overall, these data suggest that exposure to soluble Gal-9 increases NK cell degranulation and leads to Tim-3 downregulation. This observation would be consistent with a model where circulating Gal-9 mediates Tim-3 downmodulation on NK cells, leading to a contraction of the Tim-3-expressing NK cell population in chronically infected HIV-1+ individuals.

## Discussion

An emerging body of data indicates that the Tim-3/Gal-9 pathway may play a critical role in the regulation of both adaptive and innate immune mechanisms [20,21,23-29,34-38,40]. In this study, we investigated changes in the expression of the immuno-regulatory receptor Tim-3 on NK cells, and that of its ligand Gal-9 in HIV-1 infection. To our knowledge, this is the first report demonstrating loss of peripheral Tim-3+ NK cells in subjects with early and chronic untreated HIV-1 infection, with lower CD4+ T cell counts in chronically infected subjects expressing decreased percentages of Tim-3+ NK cells (Figure 1 and Additional file 1). While the observed decrease in Tim-3-expressing NK cells did not affect the CD56^bright^ NK cell subset, this specific subpopulation displayed an increased surface expression of the receptor during late primary HIV-1 infection, which occurred concomitantly with increased expression of Gal-9, predominantly by monocytes and mDCs (Figures 2 and 3). Together with previous reports, our data strongly suggest that enhanced exposure to Gal-9 during early HIV-1 infection can lead to NK cell activation, which might be beneficial to early control of HIV-1 replication (Figure 4) [20,35]. Involvement of Tim-3 in early control of HIV-1 infection and subsequent chronic exposure to Gal-9 may eventually lead to a loss of Tim-3 expression on NK cells, resulting in diminished responsiveness to its ligands.

It has been demonstrated that treatment with IL-12 and IL-18 leads to Tim-3 upregulation on CD56^bright^ NK cells, and increases the ability of this subset to produce IFN-γ in response to Gal-9 [20,21]. In line with these results, we show here that Tim-3 surface density is upregulated on peripheral CD56^bright^ NK cells during early HIV-1 infection and in untreated chronic HIV-1 infection, representing populations with high viremia. In contrast to recently published studies, we did not detect substantial IFN-γ production upon stimulation of NK cells with Gal-9 *in vitro*. However, our experiments were performed in the presence of IL-15 and IL-18 but without addition of IL-12, and the absence of this key cytokine might account for this discrepancy. Elevated inflammatory cytokine concentrations, including that of IL-12 and IL-18, have been previously described in individuals with early HIV-1 infection [43,44]. Likewise, ongoing viral replication in subjects with progressive HIV-1 infection most likely sustains a chronic inflammatory status. In these specific contexts, elevated concentrations of IL-12 and IL-18 might promote upregulation of Tim-3 on CD56^bright^ NK cells *in vivo* as observed in our cohorts of subjects with early or progressive untreated HIV-1 infection. Moreover, we observed that early HIV-1 infection is characterized by increased plasmatic levels of Gal-9. Altogether, these conditions may result in a Gal-9-induced enhancement of *in vivo* IFN-γ production by NK cells during the primary phase of the infection. Accordingly, in early HIV-1 infection, CD4+ T-cell counts negatively correlated with surface density of Tim-3 on CD56^bright^ NK cells as measured by the MFI (Additional file 3). This association between low CD4+ T-cell counts and high Tim-3 expression on CD56^bright^ NK cells may partly reflect enhanced IFN-γ-mediated elimination of HIV-1-infected CD4+ T cells. A recent study by Finney et al. also found increased expression of Tim-3 in CD56^high^ NK cells from untreated chronic subjects, yet the authors did not observe a significant decrease in percentages of Tim-3+ CD56^low^ NK cells [45]. However, low numbers of HIV-1-infected subjects included in this study, use of alternate Tim-3 reagents and particularly, differences in the NK cell classification approach for CD56^high^ and CD56^low^ NK cells may account for these distinct observations. Thus, our data suggest that increased surface density of Tim-3 on NK cells during early HIV-1 infection, followed by a loss of peripheral blood Tim-3+ subsets of cytotoxic NK cells, may be linked to progressive HIV-1 infection.

Over time, it is likely that constant exposure of Tim-3+ NK cells to Gal-9, which is more abundant in the plasma of individuals with chronic HIV-1 infection than in healthy subjects, contributes to Tim-3 downregulation. Indeed, it has been suggested that both direct and indirect stimulation can trigger internalization of activating NK cell receptors to prevent activation-induced cell death [46,47]. While it is clear that in the presence of specific cytokines, the interaction between Tim-3 and Gal-9 results in enhanced IFN-γ production by CD56^bright^ NK cells, it remains to be elucidated whether engagement of Tim-3 triggers NK cell activation and how NK cell function is modulated by changes in Tim-3 expression in the context of viral infections. Gleason and colleagues reported diminished expression of Tim-3 on NK cells from patients undergoing allogeneic hematopoietic stem cell transplantation for the treatment of leukemia, and lower expression correlated with impaired IFN-γ production, further supporting a role for Tim-3 signaling in enhancing cytokine production by NK cells [20]. In accordance with this concept, we show that engagement of Tim-3 with soluble Gal-9 triggers NK cell activation and Tim-3 downmodulation. Following exposure to Gal-9, NK cells with the most important CD107a upregulation were those with no or very low Tim-3 surface expression as a consequence of stimulation (Figure 4). These data do not directly exclude the possibility that soluble Gal-9 used to stimulate NK cells competes with the monoclonal antibody for binding to Tim-3, yet the fact that K562-activated NK cells also presented a significant decrease in surface expression of Tim-3 suggests that NK cell activation generally triggers Tim-3 downmodulation. Finally, we assessed whether NK cell activation by Gal-9 is mediated by Tim-3 by adding antibodies directed against this receptor, yet Tim-3 blockade did not significantly alter CD107a expression on NK cells, suggesting that engagement of alternate receptors by Gal-9 might contribute to this effect (Additional file 4). Accordingly, a controversially discussed publication by Leitner et al. recently suggested that Tim-3 does not act as a receptor for Gal-9 [48]. However, this study focused on Tim-3/Gal-9 interaction in T cells, and therefore caution has to be taken while extrapolating these results to NK cells. In particular, Tim-3 engagement may have distinct outcomes depending on the immune cell type expressing this receptor, suggesting that the nature of the signal mediated by Tim-3 might depend on the presence of downstream signaling molecules, which differ from one immune cell type to another. Moreover, in the manuscript by Leitner et al., stimulation of Tim-3 by Gal-9 was performed using membrane-bound and not soluble Gal-9, and this difference in methodology might impact the signal and partly explain the discrepancies between the results obtained in this setting compared to previous publications.

While further investigations are warranted to fully understand the effect of Tim-3 on NK cell function in HIV-1 infection, the permanent low expression of this potential co-activating receptor on NK cells may contribute to the previously described loss of NK cell function associated with chronic HIV-1 infection [41,42]. In line with this hypothesis, we show that CD56^neg^ NK cells, which constitute an anergic subset of NK cells that accumulates in HIV-1 infection [41,42], displayed the lowest levels of Tim-3 expression. An alternative explanation of the observed decreased percentages of peripheral blood Tim3+ CD56^dim^ NK cells in HIV-1-infected individuals could be tissue redistribution and recruitment of these highly functional cells into lymphoid tissue compartments, such as lymph nodes or mucosal sites.

Contrary to our observation in HIV-1 infection, peripheral blood and hepatic NK cells from subjects with chronic HBV infection had increased expression of Tim-3, and blockade of this receptor with an antibody directed at Tim-3 increased NK cell functions, suggesting that Tim-3, alone or in combination with other receptors, can contribute to delivering inhibitory signals [40]. It is conceivable that the NK cell surface concentration of Tim-3 determines whether the signal will be activating or inhibitory, as has been proposed for the immunoregulatory receptor 2B4 [49]. In contrast, other published data reveal that cross-linking with an anti-Tim-3 antibody abrogates cytolytic functions triggered via potent activating NK cell receptors [21]. Overall, the mechanisms underlying Tim-3 signaling appear to be highly complex and remain incompletely understood. It will therefore be critical to assess how natural ligands for Tim-3 modulate NK cell functions.

It was previously shown that individuals with chronic HIV-1 infection and high viral loads (>10,000 copies/mL) have higher levels of Gal-9 in the plasma compared to those with low viremia [35]. In our study we found that low CD4+ T cell counts and high HIV-1 viral loads were both associated with high concentrations of Gal-9 in the plasma from HIV-1-infected patients (Figure 3). Measurement of intracellular Gal-9 showed that among all lymphocyte subsets analyzed, the ligand for Tim-3 is mainly produced by monocytes and mDCs, and significantly upregulated in these cell types during early HIV-1 infection. As it has been demonstrated that Gal-9 can be both secreted and expressed at the surface of antigen presenting cells [24], it will be of paramount importance to evaluate the role of Tim-3/Gal-9 interactions in the cross-talks between NK cells and DCs, and how these impact the HIV-1-specific adaptive immune responses. It is tempting to speculate that engagement of Tim-3 in the mucosa very early in HIV-1 infection may lead to potent NK cell activation, and could limit viral spread.

## Conclusions

To conclude, while recent data indicate the existence of potential Tim-3-independent interactions between Gal-9 and NK cells [50], our study suggests that interactions between Gal-9 and Tim-3 can play a role in regulating NK cell function in HIV-1 infection. Further experiments will be required to determine how Tim-3 signaling pathways in NK cells differ from those identified in T cells and DCs so far, and how it affects NK cell function in both early and chronic infection. Overall, these data contribute to a better understanding of the innate immune mechanisms displayed in HIV-1 infection, providing a basis for further analysis into the contribution of NK cell receptors in the control of HIV-1 infection. Notably, molecules that can increase Gal-9 concentration at mucosal sites might enhance early control of HIV-1 replication, as previously suggested [35], and offer a strategy worth exploring for incorporation in future immunotherapeutic interventions or strategic HIV-1 vaccine design.

## Methods

### Study subjects (n = 98)

The study included 14 individuals identified during untreated early HIV-1 infection (Fiebig stage IV–V), 54 subjects with untreated chronic HIV-1 infection, which included 20 individuals with untreated progressive disease (median viral load, 8137; IQR, 5664–21175), 17 elite controllers with HIV-1 RNA below the limit of detection of standard viral load assays (<50 copies/mL) for at least 1 year ( among the 17 elite controller subjects studied here, viral loads were suppressed for a median of 3.5 (IQR 1.13-7.5) years), 17 viremic controllers with untreated asymptomatic HIV-1 infection (HIV-1 RNA <2000 copies/mL), 17 HIV-1–positive individuals HAART-treated with viral loads <50 copies/mL for >1 year (median, 9.1; range, 1.9-15.5), and 13 healthy HIV-1 uninfected controls (Table 1). The study was approved by the Massachusetts General Hospital Institutional Review Board, and written informed consent was obtained from all study participants.

### Flow cytometric analysis

To quantify Tim-3 expression on NK cells, cryopreserved PBMCs were thawed and stained with anti-CD3-Qdot605 (Invitrogen), anti-CD56-A700, anti-CD16-APC-Cy7 (BD Biosciences) and anti-Tim-3-PE (BioLegend, clone F38-2E2). Gating strategy is outlined in Figure 1A. Intracellular staining of Gal-9 was performed using the Fix & Perm® Medium A and B kit (Invitrogen) and anti-gal-9-PE (BioLegend, clone 9 M1-3) following surface staining with anti-CD3-Qdot605, anti-CD4-Qdot655 (Invitrogen), anti-CD56-PECy7, anti-CD16-APC-Cy7, anti-CD11c-APC, anti-CD8a-A700, anti-CD123-PECy5, anti-CD14-Pacific Blue (BD Biosciences). The LIVE/DEAD® Fixable Blue Dead Cell Stain Kit was used prior to any surface staining to exclude dead cells (Invitrogen). NK cell populations were defined as lymphocytes that were CD3-negative and included CD56^bright^ (CD3^-^CD56^+^CD16^-^), CD56^dim^ (CD3^-^CD56^+^CD16^+^), and CD56^neg^ (CD3^-^CD56^-^CD16^+^) NK cell subsets. The mDC population was defined as CD3^-^CD14^-^CD56^-^CD11c^+^CD123^-^, the pDC population as CD3^-^CD14^-^CD56^-^CD11c^-^CD123^+^, and the monocyte population as CD3^-^CD14^+^CD56^-^CD11c^+^CD123^-^ (Figure 4B). Data were acquired on LSRII 4 laser system using FacsDiva™ software version 8.8.3 (BD Biosciences). The frequency and phenotypes of NK cells, CD4+ and CD8+ T cells, monocytes, mDCs and pDCs were defined using the FlowJo™ software version 7.6.5 (Treestar, Ashland, OR).

### Quantification of Gal-9 in plasma

Plasma specimen collected at the same time points as PBMC samples were available for a subset of 82 study participants (15 elite and 16 viremic controllers, 16 individuals with chronic untreated HIV-1 infection, 15 HIV-1-positive treated individuals, 11 individuals identified during late primary HIV-1 infection, and 9 healthy HIV-1 uninfected controls). The plasma concentration of Gal-9 was quantified using an ELISA kit for soluble human Gal-9 (Uscn Life Science Inc.), according to the manufacturer’s protocol. This kit allows for the detection of Gal-9 with a dynamic range of 7.8 to 500 pg/mL.

### Flow cytometric analysis of NK cell function

NK cell activation was quantified after overnight pre-stimulation of fresh PBMCs with 1 ng/mL of IL-15 and IL-18, followed by incubation with either MHC–devoid K562 cells at an effector-to-target cell ratio of 10:1 for 6 h as previously described [51] or with 0.9 μg/mL of soluble Gal-9 (R&D) for 16 h in 1 mL of RPMI-1640 supplemented with 10% fetal bovine serum, 2 mM L-glutamine, 100 μg/ml streptomycin and 100 U/ml penicillin, 10 μl/ml anti-CD107a-PE-Cy5 antibody (BD Biosciences), 5 μg/ml brefeldin A (Sigma) and 0.3 μg/ml monensin (Golgi stop; BD Biosciences). Medium alone served as the negative control, and PMA/ionomycin (2.5 and 0.5 μg/mL, respectively) served as the positive control. PBMCs were then stained with anti-CD3-Pacific Blue, anti-CD56-PE-Cy7, anti-CD16-APC-Cy7 (BD Biosciences) and anti-Tim-3-PE (Biolegend) antibodies, fixed, permeabilized (as described above) and finally stained for intracellular IFN-γ using anti-IFN-γ-FITC antibody (BD Biosciences). Multiparameter flow cytometric analysis was performed on LSRII 4 laser instrument (BD Biosciences). The frequency and phenotypes of NK cells were defined using FlowJo™ version 7.6.5 (Treestar).

### Statistical analyses

The non-parametric Kruskal–Wallis test was used to compare phenotype frequencies and functional differences among patient groups when more than two groups were involved, and was followed by a Dunns Multiple Comparison post-test. The Mann–Whitney t test was used to compare phenotype frequencies and functional differences between two groups. The *p*-values were two-sided. Spearman’s rank correlation was used for correlation analysis. *p*-values of <0.05 were considered significant. Statistical analyses were performed using the GraphPad Prism™ software version 5.04.

## Abbreviations

NK: Natural killer; HIV-1: Human immunodeficiency virus type 1; Tim-3: T-cell immunoglobulin and mucin domain-containing molecule 3; Gal-9: Galectin-9; HAART: Highly active antiretroviral therapy; DC: Dendritic cell; HCV: Hepatitis C virus; HBV: Hepatitis B virus; IFN-γ: Interferon-gamma; IQR: Interquartile range; MFI: Mean fluorescence intensity; mDC: myeloid dendritic cell; pDC: plasmacytoid dendritic cell; PBMCs: Peripheral blood mononuclear cells; MHC: Major histocompatibility complex.

## Competing interests

The authors declare that they have no competing interests.

## Authors’ contributions

MMA, MA, SJ and UM designed experiments; UM, WG, SJ, KR and JR performed experiments; UM and SJ analyzed the data; SJ wrote the paper, MMA, MA, UM and WG edited manuscript drafts. IT and AP provided essential reagents. All authors read and approved the final manuscript.

## Supplementary Material

Additional file 1 **Percentages of Tim-3+ CD56**^**bright**^**(CD3**^**-**^**CD56**^**+**^**CD16**^**-**^**), Tim-3+ CD56**^**dim**^**(CD3**^**-**^**CD56**^**+**^**CD16**^**+**^**) and Tim-3+ CD56**^**neg**^**(CD3**^**-**^**CD56**^**-**^**CD16**^**+**^**) NK cells in 13 HIV-1 negative (HIV-) and 14 subjects with early untreated HIV-1 infection (Early), 20 untreated progressors (red, CU), 17 viremic controllers (blue, VC), 17 elite controllers (green, EC), and 17 with HAART-treated HIV-1 infection (CT).** Horizontal lines indicate the median percentages. Statistically significant difference reached when *p* < 0.05 is indicated. Click here for file

Additional file 2 **Positive correlation between the percentages of Tim-3+ CD56**^**dim **^**NK cells and CD4+ T cell counts from subjects with untreated chronic HIV-1 infection and available clinical data (matching the day of PBMCs and plasma sample collection), including 17 elite controllers (green), 17 viremic controllers (blue) and 17 individuals with untreated chronic progressive infection (red).** Click here for file

Additional file 3 **Inverse correlation between the MFI of Tim-3 on CD56**^**bright **^**NK cells and CD4+ T cell counts in 9 subjects with early HIV-1 infection and available clinical data matching the date of the PBMCs sample used for the quantification of Tim-3 on NK cells.** Click here for file

Additional file 4 **Downregulation of Tim-3 (upper graph) and upregulation of CD107a (lower graph) on NK cells stimulated with Gal-9 in the presence of Tim-3 blocking antibodies.** PBMCs from 3 healthy individuals were incubated overnight in the presence of 1 ng/mL of IL-12 and 10 ng/mL of IL-18 prior to incubation with Gal-9 (1 ug/mL) or K562 cells (effector:target ratio of 10:1) in the presence or absence of anti-Tim-3 antibodies (20 ug/mL) for 6 h as indicated. Lactose (20 mM final) was used as a control for Gal-9 blockade. Click here for file

## References

[B1] 2012 UNAIDS Report on the Global AIDS Epidemic http://www.unaids.org/en/media/unaids/contentassets/documents/epidemiology/2012/gr2012/20121120_UNAIDS_Global_Report_2012_en.pdf

[B2] Martin MP Gao X Lee JH Nelson GW Detels R Goedert JJ Buchbinder S Hoots K Vlahov D Trowsdale J Epistatic interaction between KIR3DS1 and HLA-B delays the progression to AIDS Nat Genet 2002 31 429 434 12134147 10.1038/ng934

[B3] Martin MP Qi Y Gao X Yamada E Martin JN Pereyra F Colombo S Brown EE Shupert WL Phair J Innate partnership of HLA-B and KIR3DL1 subtypes against HIV-1 Nat Genet 2007 39 733 740 10.1038/ng2035 17496894 PMC4135476

[B4] Alter G Martin MP Teigen N Carr WH Suscovich TJ Schneidewind A Streeck H Waring M Meier A Brander C Differential natural killer cell-mediated inhibition of HIV-1 replication based on distinct KIR/HLA subtypes J Exp Med 2007 204 3027 3036 10.1084/jem.20070695 18025129 PMC2118524

[B5] Alter G Rihn S Walter K Nolting A Martin M Rosenberg ES Miller JS Carrington M Altfeld M HLA class I subtype-dependent expansion of KIR3DS1+ and KIR3DL1+ NK cells during acute human immunodeficiency virus type 1 infection J Virol 2009 83 6798 6805 10.1128/JVI.00256-09 19386717 PMC2698561

[B6] Boulet S Kleyman M Kim JY Kamya P Sharafi S Simic N Bruneau J Routy JP Tsoukas CM Bernard NF A combined genotype of KIR3DL1 high expressing alleles and HLA-B*57 is associated with a reduced risk of HIV infection AIDS 2008 22 1487 1491 10.1097/QAD.0b013e3282ffde7e 18614872

[B7] Boulet S Sharafi S Simic N Bruneau J Routy JP Tsoukas CM Bernard NF Increased proportion of KIR3DS1 homozygotes in HIV-exposed uninfected individuals AIDS 2008 22 595 599 10.1097/QAD.0b013e3282f56b23 18317000

[B8] Boulet S Song R Kamya P Bruneau J Shoukry NH Tsoukas CM Bernard NF HIV protective KIR3DL1 and HLA-B genotypes influence NK cell function following stimulation with HLA-devoid cells J Immunol 2010 184 2057 2064 10.4049/jimmunol.0902621 20061407

[B9] Parsons MS Boulet S Song R Bruneau J Shoukry NH Routy JP Tsoukas CM Bernard NF Mind the gap: lack of association between KIR3DL1*004/HLA-Bw4-induced natural killer cell function and protection from HIV infection J Infect Dis 2010 202 Suppl 3 S356 360 20887224 10.1086/655966

[B10] Alter G Heckerman D Schneidewind A Fadda L Kadie CM Carlson JM Oniangue-Ndza C Martin M Li B Khakoo SI HIV-1 adaptation to NK-cell-mediated immune pressure Nature 2011 476 96 100 10.1038/nature10237 21814282 PMC3194000

[B11] Strbo N de Armas L Liu H Kolber MA Lichtenheld M Pahwa S IL-21 augments natural killer effector functions in chronically HIV-infected individuals AIDS 2008 22 1551 1560 10.1097/QAD.0b013e3283089367 18670213 PMC2665999

[B12] O'Connell KA Han Y Williams TM Siliciano RF Blankson JN Role of natural killer cells in a cohort of elite suppressors: low frequency of the protective KIR3DS1 allele and limited inhibition of human immunodeficiency virus type 1 replication in vitro J Virol 2009 83 5028 5034 10.1128/JVI.02551-08 19211742 PMC2682110

[B13] Paust S von Andrian UH Natural killer cell memory Nat Immunol 2011 12 500 508 21739673 10.1038/ni.2032

[B14] Andrews DM Estcourt MJ Andoniou CE Wikstrom ME Khong A Voigt V Fleming P Tabarias H Hill GR van der Most RG Innate immunity defines the capacity of antiviral T cells to limit persistent infection J Exp Med 2010 207 1333 1343 10.1084/jem.20091193 20513749 PMC2882831

[B15] Cooper MA Fehniger TA Fuchs A Colonna M Caligiuri MA NK cell and DC interactions Trends Immunol 2004 25 47 52 10.1016/j.it.2003.10.012 14698284

[B16] Robbins SH Bessou G Cornillon A Zucchini N Rupp B Ruzsics Z Sacher T Tomasello E Vivier E Koszinowski UH Dalod M Natural killer cells promote early CD8 T cell responses against cytomegalovirus PLoS Pathog 2007 3 e123 10.1371/journal.ppat.0030123 17722980 PMC1950948

[B17] Waggoner SN Cornberg M Selin LK Welsh RM Natural killer cells act as rheostats modulating antiviral T cells Nature 2012 481 394 398 22101430 10.1038/nature10624 PMC3539796

[B18] Lanier LL NK cell recognition Annu Rev Immunol 2005 23 225 274 10.1146/annurev.immunol.23.021704.115526 15771571

[B19] Khademi M Illes Z Gielen AW Marta M Takazawa N Baecher-Allan C Brundin L Hannerz J Martin C Harris RA T Cell Ig- and mucin-domain-containing molecule-3 (TIM-3) and TIM-1 molecules are differentially expressed on human Th1 and Th2 cells and in cerebrospinal fluid-derived mononuclear cells in multiple sclerosis J Immunol 2004 172 7169 7176 15153541 10.4049/jimmunol.172.11.7169

[B20] Gleason MK Lenvik TR McCullar V Felices M O'Brien MS Cooley SA Verneris MR Cichocki F Holman CJ Panoskaltsis-Mortari A Tim-3 is an inducible human natural killer cell receptor that enhances interferon gamma production in response to galectin-9 Blood 2012 119 3064 3072 10.1182/blood-2011-06-360321 22323453 PMC3321868

[B21] Ndhlovu LC Lopez-Verges S Barbour JD Jones RB Jha AR Long BR Schoeffler EC Fujita T Nixon DF Lanier LL Tim-3 marks human natural killer cell maturation and suppresses cell-mediated cytotoxicity Blood 2012 119 3734 3743 10.1182/blood-2011-11-392951 22383801 PMC3335380

[B22] Zhu C Anderson AC Kuchroo VK TIM-3 and its regulatory role in immune responses Curr Top Microbiol Immunol 2011 350 1 15 20700701 10.1007/82_2010_84

[B23] Monney L Sabatos CA Gaglia JL Ryu A Waldner H Chernova T Manning S Greenfield EA Coyle AJ Sobel RA Th1-specific cell surface protein Tim-3 regulates macrophage activation and severity of an autoimmune disease Nature 2002 415 536 541 10.1038/415536a 11823861

[B24] Li H Wu K Tao K Chen L Zheng Q Lu X Liu J Shi L Liu C Wang G Zou W Tim-3/galectin-9 signaling pathway mediates T-cell dysfunction and predicts poor prognosis in patients with hepatitis B virus-associated hepatocellular carcinoma Hepatology 2012 56 1342 1351 10.1002/hep.25777 22505239

[B25] Jones RB Ndhlovu LC Barbour JD Sheth PM Jha AR Long BR Wong JC Satkunarajah M Schweneker M Chapman JM Tim-3 expression defines a novel population of dysfunctional T cells with highly elevated frequencies in progressive HIV-1 infection J Exp Med 2008 205 2763 2779 10.1084/jem.20081398 19001139 PMC2585847

[B26] Sakhdari A Mujib S Vali B Yue FY Macparland S Clayton K Jones RB Liu J Lee EY Benko E Tim-3 Negatively Regulates Cytotoxicity in Exhausted CD8(+) T Cells in HIV Infection PLoS One 2012 7 e40146 10.1371/journal.pone.0040146 22792231 PMC3390352

[B27] Golden-Mason L Palmer BE Kassam N Townshend-Bulson L Livingston S McMahon BJ Castelblanco N Kuchroo V Gretch DR Rosen HR Negative immune regulator Tim-3 is overexpressed on T cells in hepatitis C virus infection and its blockade rescues dysfunctional CD4+ and CD8+ T cells J Virol 2009 83 9122 9130 10.1128/JVI.00639-09 19587053 PMC2738247

[B28] Vali B Jones RB Sakhdari A Sheth PM Clayton K Yue FY Gyenes G Wong D Klein MB Saeed S HCV-specific T cells in HCV/HIV co-infection show elevated frequencies of dual Tim-3/PD-1 expression that correlate with liver disease progression Eur J Immunol 2010 40 2493 2505 10.1002/eji.201040340 20623550

[B29] Zhu C Anderson AC Schubart A Xiong H Imitola J Khoury SJ Zheng XX Strom TB Kuchroo VK The Tim-3 ligand galectin-9 negatively regulates T helper type 1 immunity Nat Immunol 2005 6 1245 1252 10.1038/ni1271 16286920

[B30] DeKruyff RH Bu X Ballesteros A Santiago C Chim YL Lee HH Karisola P Pichavant M Kaplan GG Umetsu DT T cell/transmembrane, Ig, and mucin-3 allelic variants differentially recognize phosphatidylserine and mediate phagocytosis of apoptotic cells J Immunol 2010 184 1918 1930 10.4049/jimmunol.0903059 20083673 PMC3128800

[B31] Nakayama M Akiba H Takeda K Kojima Y Hashiguchi M Azuma M Yagita H Okumura K Tim-3 mediates phagocytosis of apoptotic cells and cross-presentation Blood 2009 113 3821 3830 10.1182/blood-2008-10-185884 19224762

[B32] Chiba S Baghdadi M Akiba H Yoshiyama H Kinoshita I Dosaka-Akita H Fujioka Y Ohba Y Gorman JV Colgan JD Tumor-infiltrating DCs suppress nucleic acid-mediated innate immune responses through interactions between the receptor TIM-3 and the alarmin HMGB1 Nat Immunol 2012 13 832 842 10.1038/ni.2376 22842346 PMC3622453

[B33] Wada J Kanwar YS Identification and characterization of galectin-9, a novel beta-galactoside-binding mammalian lectin J Biol Chem 1997 272 6078 6086 10.1074/jbc.272.9.6078 9038233

[B34] Bi S Earl LA Jacobs L Baum LG Structural features of galectin-9 and galectin-1 that determine distinct T cell death pathways J Biol Chem 2008 283 12248 12258 10.1074/jbc.M800523200 18258591 PMC2431002

[B35] Elahi S Niki T Hirashima M Horton H Galectin-9 binding to Tim-3 renders activated human CD4+ T cells less susceptible to HIV-1 infection Blood 2012 119 4192 4204 10.1182/blood-2011-11-389585 22438246 PMC3359739

[B36] Anderson AC Anderson DE Bregoli L Hastings WD Kassam N Lei C Chandwaskar R Karman J Su EW Hirashima M Promotion of tissue inflammation by the immune receptor Tim-3 expressed on innate immune cells Science 2007 318 1141 1143 10.1126/science.1148536 18006747

[B37] Sabatos CA Chakravarti S Cha E Schubart A Sanchez-Fueyo A Zheng XX Coyle AJ Strom TB Freeman GJ Kuchroo VK Interaction of Tim-3 and Tim-3 ligand regulates T helper type 1 responses and induction of peripheral tolerance Nat Immunol 2003 4 1102 1110 10.1038/ni988 14556006

[B38] Bi S Hong PW Lee B Baum LG Galectin-9 binding to cell surface protein disulfide isomerase regulates the redox environment to enhance T-cell migration and HIV entry Proc Natl Acad Sci USA 2011 108 10650 10655 10.1073/pnas.1017954108 21670307 PMC3127870

[B39] Cao E Zang X Ramagopal UA Mukhopadhaya A Fedorov A Fedorov E Zencheck WD Lary JW Cole JL Deng H T cell immunoglobulin mucin-3 crystal structure reveals a galectin-9-independent ligand-binding surface Immunity 2007 26 311 321 10.1016/j.immuni.2007.01.016 17363302

[B40] Ju Y Hou N Meng J Wang X Zhang X Zhao D Liu Y Zhu F Zhang L Sun W T cell immunoglobulin- and mucin-domain-containing molecule-3 (Tim-3) mediates natural killer cell suppression in chronic hepatitis B J Hepatol 2010 52 322 329 10.1016/j.jhep.2009.12.005 20133006

[B41] Alter G Teigen N Davis BT Addo MM Suscovich TJ Waring MT Streeck H Johnston MN Staller KD Zaman MT Sequential deregulation of NK cell subset distribution and function starting in acute HIV-1 infection Blood 2005 106 3366 3369 10.1182/blood-2005-03-1100 16002429

[B42] Mavilio D Lombardo G Benjamin J Kim D Follman D Marcenaro E O'Shea MA Kinter A Kovacs C Moretta A Fauci AS Characterization of CD56-/CD16+ natural killer (NK) cells: a highly dysfunctional NK subset expanded in HIV-infected viremic individuals Proc Natl Acad Sci USA 2005 102 2886 2891 10.1073/pnas.0409872102 15699323 PMC549494

[B43] Gay C Dibben O Anderson JA Stacey A Mayo AJ Norris PJ Kuruc JD Salazar-Gonzalez JF Li H Keele BF Cross-sectional detection of acute HIV infection: timing of transmission, inflammation and antiretroviral therapy PLoS One 2011 6 e19617 10.1371/journal.pone.0019617 21573003 PMC3091862

[B44] Stacey AR Norris PJ Qin L Haygreen EA Taylor E Heitman J Lebedeva M DeCamp A Li D Grove D Induction of a striking systemic cytokine cascade prior to peak viremia in acute human immunodeficiency virus type 1 infection, in contrast to more modest and delayed responses in acute hepatitis B and C virus infections J Virol 2009 83 3719 3733 10.1128/JVI.01844-08 19176632 PMC2663284

[B45] Finney CA Ayi K Wasmuth JD Sheth PM Kaul R Loutfy M Kain KC Serghides L HIV Infection Deregulates Tim-3 Expression on Innate Cells; Combination Antiretroviral Therapy Results in Partial Restoration J Acquir Immune Defic Syndr 2013 63 161 167 10.1097/QAI.0b013e318285cf13 23314411

[B46] Alter G Jost S Rihn S Reyor L Nolan B Ghebremichael M Bosch R Altfeld M Lauer G Reduced frequencies of NKp30 + NKp46+, CD161+ and NKG2D + NK cells in acute HCV infection may predict viral clearance J Hepatol 2010 10.1016/j.jhep.2010.11.030 PMC3729214 21168454

[B47] Lanier LL Up on the tightrope: natural killer cell activation and inhibition Nat Immunol 2008 9 495 502 18425106 10.1038/ni1581 PMC2669298

[B48] Leitner J Rieger A Pickl WF Zlabinger G Grabmeier-Pfistershammer K Steinberger P TIM-3 Does Not Act as a Receptor for Galectin-9 PLoS Pathog 2013 9 e1003253 10.1371/journal.ppat.1003253 23555261 PMC3605152

[B49] Chlewicki LK Velikovsky CA Balakrishnan V Mariuzza RA Kumar V Molecular basis of the dual functions of 2B4 (CD244) J Immunol 2008 180 8159 8167 18523281 10.4049/jimmunol.180.12.8159

[B50] Golden-Mason L McMahan RH Strong M Reisdorph R Mahaffey S Palmer BE Cheng L Kulesza C Hirashima M Niki T Rosen HR Galectin-9 functionally impairs natural killer (NK) cells in humans and mice J Virol 2013 87 4835 4845 10.1128/JVI.01085-12 23408620 PMC3624298

[B51] Alter G Malenfant JM Altfeld M CD107a as a functional marker for the identification of natural killer cell activity J Immunol Methods 2004 294 15 22 10.1016/j.jim.2004.08.008 15604012

