# Supplementary figures and images for: Dysregulated Tim-3 expression on natural killer cells is associated with increased Galectin-9 levels in HIV-1 infection
Source: Retrovirology. 2013 Jul 18;10:74. doi: 10.1186/1742-4690-10-74 (PMC3750478; doi:10.1186/1742-4690-10-74)

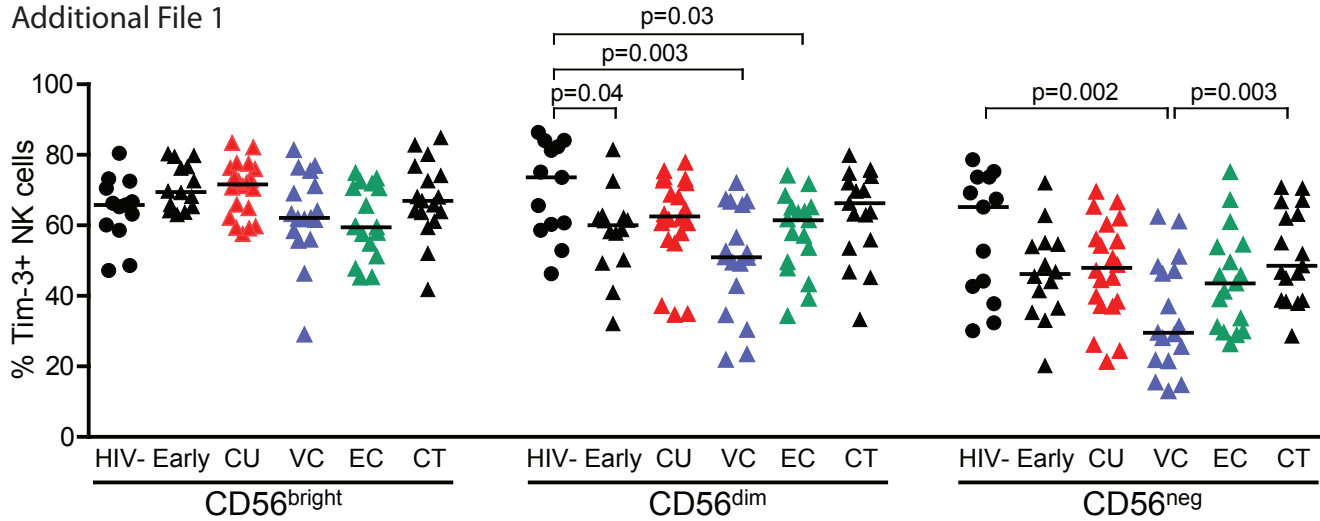

Supplement: Additional file 1 — Percentages of Tim-3+ CD56bright(CD3-CD56+CD16-), Tim-3+ CD56dim(CD3-CD56+CD16+) and Tim-3+ CD56neg(CD3-CD56-CD16+) NK cells in 13 HIV-1 negative (HIV-) and 14 subjects with early untreated HIV-1 infection (Early), 20 untreated progressors (red, CU), 17 viremic controllers (blue, VC), 17 elite controllers (green, EC), and 17 with HAART-treated HIV-1 infection (CT). Horizontal lines indicate the median percentages. Statistically significant difference reached when p < 0.05 is indicated. [file 1742-4690-10-74-S1.pdf]

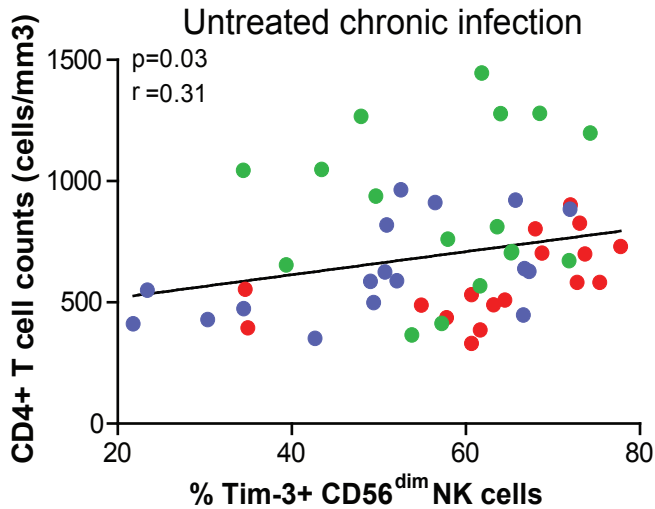

Supplement: Additional file 2 — Positive correlation between the percentages of Tim-3+ CD56dim NK cells and CD4+ T cell counts from subjects with untreated chronic HIV-1 infection and available clinical data (matching the day of PBMCs and plasma sample collection), including 17 elite controllers (green), 17 viremic controllers (blue) and 17 individuals with untreated chronic progressive infection (red). [file 1742-4690-10-74-S2.pdf]

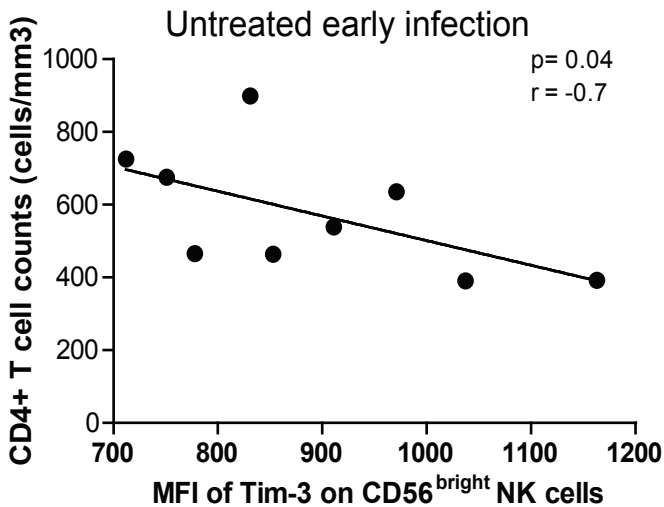

Supplement: Additional file 3 — Inverse correlation between the MFI of Tim-3 on CD56bright NK cells and CD4+ T cell counts in 9 subjects with early HIV-1 infection and available clinical data matching the date of the PBMCs sample used for the quantification of Tim-3 on NK cells. [file 1742-4690-10-74-S3.pdf]

# Additional file 4

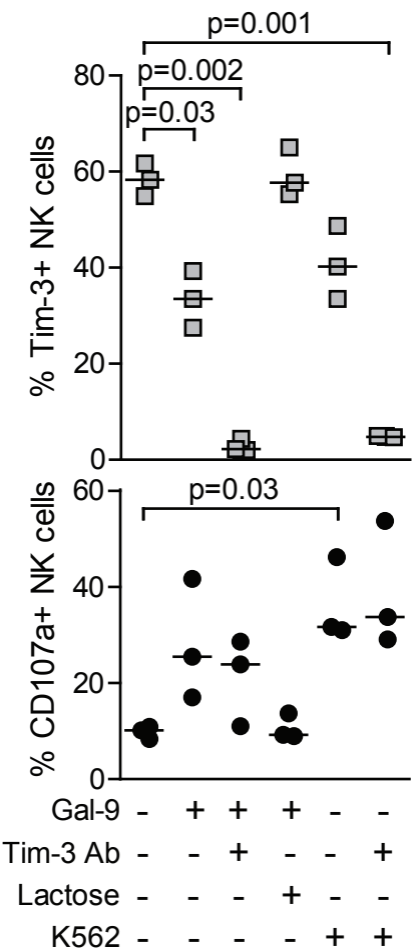

Supplement: Additional file 4 — Downregulation of Tim-3 (upper graph) and upregulation of CD107a (lower graph) on NK cells stimulated with Gal-9 in the presence of Tim-3 blocking antibodies. PBMCs from 3 healthy individuals were incubated overnight in the presence of 1 ng/mL of IL-12 and 10 ng/mL of IL-18 prior to incubation with Gal-9 (1 ug/mL) or K562 cells (effector:target ratio of 10:1) in the presence or absence of anti-Tim-3 antibodies (20 ug/mL) for 6 h as indicated. Lactose (20 mM final) was used as a control for Gal-9 blockade. [file 1742-4690-10-74-S4.pdf]
